# Isotropic and Anisotropic Complex Refractive Index of PEDOT:PSS

**DOI:** 10.3390/polym15153298

**Published:** 2023-08-04

**Authors:** Lara Velasco Davoise, Rafael Peña Capilla, Ana M. Díez-Pascual

**Affiliations:** 1 Universidad de Alcalá, Facultad de Ciencias, Departamento de Química Analítica, Química Física e Ingeniería Química, Ctra. Madrid-Barcelona Km. 33.6, 28805 Alcalá de Henares, Madrid, Spain; lara.velasco@uah.es; 2 Universidad de Alcalá, Departamento de Teoría de la Señal y Comunicaciones, Ctra. Madrid-Barcelona Km. 33.6, 28805 Alcalá de Henares, Madrid, Spain; rafael.pena@uah.es

**Keywords:** PEDOT:PSS, refractive index, extinction coefficient, modelling

## Abstract

In this work, the complex refractive indexes of seven PEDOT:PSS samples, three with isotropic behavior and four with optical anisotropy, were determined. For the anisotropic samples, the ordinary and extraordinary components of the refractive index were described. The effect of the film thickness, measurement technique and preparation method on the extinction coefficient (*k*) and refractive index (*n*) of each sample was also discussed. Important differences (up to 20% in the average *n*) were found among the samples investigated. In most anisotropic films, the mean value of the extraordinary component was between 7 and 10% higher than that of the ordinary. In the three isotropic films, the average *k* rose when the film thickness increased. Moreover, the different sets of refractive index data were fitted to three different models: the original Forouhi–Bloomer model, the Liu (2007) model and the revised version of the Forouhi–Bloomer model (2019). In general, Liu’s model gave better results, with small errors in *n* and *k* (<7.81 and 4.68%, respectively, in all the cases). However, this model had seven fitting parameters, which led to significantly longer computation time than the other two models. The influence of the differences in the measurement of the complex refractive index on the simulation of the optical properties of PEDOT:PSS multilayers was discussed. The results showed that *n* must be known precisely to accurately calculate the light absorption in a multilayer, without ignoring the isotropic or anisotropic behavior of the material or the influence of the layer thickness on its optical properties. This study aids in the development of simulation and optimization tools that allow understanding the optical properties of PEDOT:PSS films for their potential applications in organic optoelectronic devices, such as organic solar cells.

## 1. Introduction

Currently, great attention is being directed towards photovoltaics since it is a clean, inexhaustible and cheap energy technology. In particular, organic solar cells (OSCs) are attracting much interest from both academia and industry due to the important advantages they show compared to silicon-based devices such as their lightweight, flexibility, lower processing costs and lower environmental impact [1,2]. The performance of OSCs greatly depends on the properties of the interfaces between the photoactive layers and the electrodes [3]. Therefore, hole transport layers (HTLs) and electron transport layers (ETLs) are used in order to allow energy level alignment between the active layers and the electrodes, improving the optical field distribution, blocking excitons and reducing surface recombination [3].

Poly(3,4-ethylenedioxythiophene) (PEDOT) is a conducting polymer with conjugated double bonds regarded as a promising material for organic optoelectronic devices due to its high and stable electrical conductivity, high work function and optical transparency (transmittance higher than 90% in the visible range). It enables cost-effective and flexible devices as well as roll-to-roll mass production. PEDOT, which is insoluble in most solvents, can be dispersed in water by using poly(styrene sulfonate) (PSS) as a counter ion, which also serves as an excellent oxidizing agent, charge compensator and as a template for polymerization [4].

PEDOT:PSS is a polymeric mixture of two ionomers that shows high electrical conductivity (from 735 S cm^−1^ up to 1418 S cm^−1^ [4]), high work function (5.1 eV [5]), optical transparency in the visible range (transmittance about 90% [6]), high mechanical flexibility and excellent thermal stability [7]. In addition, PEDOT:PSS has the highest efficiency for transforming heat into electricity among conductive organic thermoelectric materials. Its figure of merit $ZT=S^{2}\sigma T/\kappa$, which is calculated from the Seebeck coefficient (*S*), the electrical conductivity (*σ*) and the thermal conductivity (κ) reach a value of 0.42 [8]. Its electrical conductivity can be further improved via addition of organic compounds or solvents with a high boiling point such as methylpyrrolidone, dimethyl sulfoxide, ionic liquids and surfactants, reaching values as high as 4600 S cm^−1^ [9]. It also exhibits good ductility and high stretchability prior to fracture and self-healing properties when submerged in water after sustaining mechanical damage [10]. All these features make PEDOT:PSS suitable as a HTL in OSCs [3,11]. In fact, it has been one of the most used materials for this purpose for years [3]. In addition, PEDOT:PSS has been proposed as an alternative to indium tin oxide (ITO) [4,12], the most popular material as a transparent electrode. Thus, some of the drawbacks related to the use of ITO would be solved, such as the scarcity of indium, its expensiveness and high mechanical brittleness that affects its application in flexible devices and its poor adhesion to organic and polymeric materials [13].

A comprehensive knowledge of the refractive index (*n*) of a material and the factors that influence this important parameter is crucial to evaluate its possible applications in optoelectronic devices such as liquid crystal displays (LCDs), light-emitting diodes (LEDs), solar cells, touch panel displays, lasers, detectors, optical fibers, etc. [7,14]. Moreover, the opto-electronic properties are of great importance for applications in material science, medicine, chemical analysis, light propagation modeling in turbid media, and so forth. In particular, the complex refractive index should be precisely known in order to develop simulation and optimization tools. Among these, the scattering matrix method (SMM) is a fundamental tool for the design of solar cells, LEDs, anti-reflective coatings (ARCs) in various optical systems, etc. [15,16]. For instance, the SMM has been applied to the design of ARCs in high efficiency multi-junction solar cells [15], in order to achieve good anti-reflection properties over a wide absorption spectrum. The SMM has also been successfully applied to the simulation of OSCs [16,17]. Hence, the development of accurate and relatively simple methods to calculate *n* is of a great interest.

In the literature, numerous studies have been reported on the modelling of the optical properties of PEDOT:PSS and other materials used for the manufacture of OSCs, including the complex refractive index [12,18,19,20,21,22,23]. However, in most of the studies reported, the optical properties of thin layers of these materials have been considered to be isotropic, while generally they do not fulfill this condition [19]. This is particularly important in OSCs, because in thin films of conjugated polymers, the macroscopic optical response depends both on the intrinsic electronic structure of the chain and on the inter-molecular interactions determined by their relative orientation and position. Therefore, the isotropic or anisotropic optical behavior of thin polymeric films depends on the sample preparation method. For instance, spin-coated thin films have been shown to be uniaxial anisotropic with the optic axis parallel to the surface normal of the film [19]. In this case, the linear optical response is described by the principal indices of refraction, the ordinary complex index of refraction (∥, parallel to the surface plane of the film) and the extraordinary complex index of refraction (⊥, perpendicular to the surface plane of the film). Also, the complex refractive index is strongly dependent on the measurement method, as will be described in a following section.

In this regard, the main aim of this work is to describe the complex refractive index of seven PEDOT:PSS samples, three with isotropic optical behavior and four with anisotropy. For the anisotropic, the ordinary and extraordinary components of the refractive index are shown. The influence of the film thickness, measurement method and preparation method on the extinction coefficient (*k*) and *n* of each sample is discussed. In addition, the different sets of refractive index data were fitted to three dispersion formulae, the original Forouhi–Bloomer model [24], the Liu (2007) model [25] and the revised version of the Forouhi–Bloomer model (2019) [26]. These models are supported by the quantum theory of absorption, provide accurate fittings for *n* and *k* and, simultaneously, enable to estimate the energy bandgap. These features make them more suitable than other commonly used models, such as Sellmeier or Cauchy equations [24].

Finally, the influence of the differences in the measurements of the complex refractive index on the simulation of the optical properties of PEDOT:PSS multilayers is discussed. The results show that to accurately calculate the light absorption in a multilayer, the refractive index should be known precisely, without ignoring the isotropic or anisotropic behavior of the material or the influence of the layer thickness on its optical properties.

## 2. Practical Considerations Related to the Determination of the Complex Refractive Index of a Thin Film

In the following sections, some practical aspects that have strong influence on the measurement of the refractive index of a thin film are discussed. Firstly, the main deposition methods of PEDOT:PSS thin films are briefly described. Then, the main measurement methods of the complex refractive index of these films are shown. Finally, a brief overview of the original Forouhi–Bloomer equations and other related fitting models, which will be applied in this work to fit the experimental values of the complex refractive index of the PEDOT:PSS films, is provided.

### 2.1. Preparation Methods

The optical properties of PEDOT:PSS including *n* and *k* are strongly dependent on the film preparation method. Four main approaches have been reported to prepare PEDOT:PSS films, as indicated below.

Spin coating is used to deposit thin films onto flat substrates. On the center of the substrate, a small amount of the coating material is applied. The substrate is then rotated at a certain speed to spread the material by centrifugal force. Rotation is continued while the fluid spins off the edges of the substrate, until the desired thickness of the film is achieved. The higher the angular speed of spinning, the thinner the film. The thickness of the film also depends on the viscosity and concentration of the solution [27]. Spin coating yields thin films with a relatively planar surface in a wide range of thicknesses (from nanometers to microns [28]). This method causes an axial symmetry in the material that leads to optical anisotropy. This has previously been shown for thin films of conjugated polymers, with uniaxial anisotropy arising from a preferential orientation of the conjugated polymer with the chains primarily in the surface plane [19].

Capillary force molding (CFM) is a simple, scalable and inexpensive method for fabricating nanometer scale patterns with high resolution [18], suitable to develop flexible substrates. Nanomolding allows the fabrication of nanostructures with inherently controlled size and geometry. Therefore, samples prepared with CFM have uniform thickness [29]. In addition, no optical anisotropies have been observed in these samples.

Spiral bar coating consists of placing an excess of a solution containing the material to be deposited on a substrate and spreading it with the aid of a bar that has a wire spiraling around it [28]. The gap between the wire and the substrate determines the amount of solution allowed to go through, hence the film thickness. This method can form uniform films on rigid or flexible substrates over large areas [28]. According to Syvory et al. [22], the roughness of the layers prepared with this method ranges between 2.5% and 4%. No optical anisotropies have been reported for samples prepared using this approach.

Electrospray deposition is a process that utilizes the balance of electrostatic forces and surface tension within a charged spray to produce charged microdroplets with a narrow dispersion in size [30]. These droplets are uniformly dispersed over a substrate to produce nanostructured coatings. The charge, size and motion of the droplets can be controlled by electrical means with high accuracy. Therefore, the films prepared via electrospray deposition have uniform thickness. However, uniaxial anisotropy has been found in these samples. According to Ino et al. [31], it probably arises from a geometric anisotropy of the polymer chains, due to a preferential molecular orientation during the film growth.

### 2.2. Measurement Methods

Currently, there are several methods to measure the refractive index of thin films, including ellipsometry, refractometry and optical spectrometry. The measurement methods must take into account the possible anisotropy of the material. An optically isotropic material is that in which the complex refractive index is the same in all directions. In contrast, optically anisotropic materials have different propagation properties when light passes through them in different directions. In materials with uniaxial anisotropy, there is a single direction, the optic axis, governing the optical anisotropy. All directions perpendicular to it are optically equivalent. Thus, rotating the material around the axis does not change its optical behavior. A light beam propagating parallel to the optic axis is governed by the ordinary refractive index, *n_o_*. Light rays propagating at an angle to the optic axis are governed by a variable refractive index, between *n_o_* and the so-called extraordinary refractive index, *n_e_*, depending on the incidence angle of the ray [22].

Spectroscopic ellipsometry (SE) is an indirect optical characterization method used to obtain the dielectric properties (complex refractive index or dielectric function) of thin films. It is a non-destructive and contactless technique based on the change in the polarization state of light as it is reflected obliquely from a thin film sample [22]. Standard ellipsometry is applied when no *s* polarized light (in which the electric field is normal to the plane of incidence) is converted into *p* polarized light (in which the electric field is along the plane of incidence) nor vice versa. This occurs in optically isotropic samples. Standard ellipsometry can also be used for optically uniaxial samples when the optical axis is aligned parallel to the surface normal. When *s* polarized light is converted into *p* polarized light or vice versa, variable angle spectroscopic ellipsometry (VASE) should be used. For instance, optically uniaxial samples or optically biaxial samples in which the optic axis is not aligned parallel to the surface normal should be characterized by means of VASE.

Other techniques for measuring the complex refractive index are refractometry, which measures the extent to which light is bent when it moves from air into a sample, typically liquid, and optical spectrometry, which measures the reflectance and transmittance of the film and relates them to the complex refractive index.

### 2.3. The Forouhi–Bloomer Equations and Other Related Fitting Models

The Forouhi–Bloomer model (FB) is a mathematical formulation implemented in 1986 used to fit the real and imaginary parts of the complex refractive index of amorphous or crystalline materials. The equations of this model are consistent with Kramers–Kronig analysis. In materials with just one peak of absorption, the extinction coefficient can be expressed as [24]:

(1) $$k\left(E\right)=\{\begin{array}{c} \frac{A\cdot {\left(E-E_{g}\right)}^{2}}{E^{2}-B\cdot E+C};\;forE>Eg\\ 0;\;forE\leq Eg \end{array}$$

and the refractive index as:

(2) $$n\left(E\right)=\sqrt{\varepsilon_{\infty }}+\frac{B_{0}\cdot E+C_{0}}{E^{2}-B\cdot E+C}$$

where the relations between the coefficients are:

(3) $$B_{0}=\frac{A}{Q}\cdot \left(\frac{-B^{2}}{2}+B\cdot E_{g}-E_{g}^{2}+C\right)$$

(4) $$C_{0}=\frac{A}{Q}\cdot \left(\left(E_{g}^{2}+C\right)\cdot \frac{B}{2}-2\cdot E_{g}\cdot C\right)$$

(5) $$Q=\frac{1}{2}\cdot \sqrt{4\cdot C-B^{2}}$$

Consequently, *n* and *k* can be modeled using just five parameters. *A*, *B* and *C* are positive non-zero parameters referring to the electronic structure of the material. *A* is a dimensionless parameter related to the dipole matrix squared and gives the amplitude of *k* peak. Generally, 0 < *A* < 2. *B*/2 (in eV) is approximately the energy at which *k* is maximum, i.e., the energy at which the peak of absorption is reached. As the value of *B* increases, the absorption peak is shifted towards the UV region. Generally, 3 < *B* < 30. The third parameter, *C* (in eV^2^), is related to the broadening term of the absorption peak. The higher the *C* value is, the higher the absorption peak but the smaller its amplitude is. Generally, 3 < *C* < 150. The term $\varepsilon_{\infty }$ (in eV) is an additional parameter corresponding to the high energy dielectric constant. It is at least superior to one and equal to the dielectric function when *E*→ꝏ. Finally, *E_g_* (in eV) is the energy band gap, that is, the minimum energy required for a transition from the valence band to the conduction band.

Although this model has important advantages over older semi-empirical models, such as Cauchy equations or Sellmeier equations [32], which are either valid only over a limited spectral range, or do not accurately fit experimental data, it still does not consider non-parabolic conduction and valence bands. In addition, the FB model assumes that the absorption becomes zero below the energy band gap. However, in practice, semiconductors and dielectrics are known to experience an exponential decay of the absorption coefficient (α) below the bandgap, which is called the Urbach tail [33]. Therefore, different works proposed some modifications of the original FB model, as described below. The term *A*(*E* − *E_g_*)^2^ in the numerator of Equation (1), known as the *θ* parameter, is proportional to the number of possible transitions from the valence band to the conduction band [25].

(6) $$\Theta =A\cdot {\left(E-E_{g}\right)}^{2}$$

In the FB model, the valence and conduction bands are assumed to be parabolic and that no electronic state exists in the optical band gap. Further, it does not consider phonon effects. Thus, it cannot accurately describe the absorption processes of many semiconductor materials. The latest modifications of this model made by McGahan et al. [34] and Liu et al. [25] changed the definition of the *θ* parameter to make the model more complete. McGahan and coworkers rewrote *θ* as follows:

(7) $$\Theta =A_{1}{\left(E-E_{g}\right)}^{2}+A_{2}{\left(E-E_{g}\right)}^{3/2}$$

This expression yields different equations for *n* and *k*. However, the form of the formulas reported by McGahan et al. [34] is very complicated, since they need to be solved using numerical integration. To more accurately and easily describe the dispersion of the optical constants of thin films, a different correction was proposed by Liu et al. [25]. In the new model, the Θ parameter was written as the θ(E)’s second Taylor series in the (E − E_g_):

(8) $$\Theta =A+B\left(E-E_{g}\right)+C{\left(E-E_{g}\right)}^{2}$$

Hence, Liu et al. [25] obtained the following expressions for *k* and *n*:

(9) $$k\left(E\right)=\frac{A+B\left(E-E_{g}\right)+C{\left(E-E_{g}\right)}^{2}}{E^{2}-D\cdot E+F}$$

(10) $$n\left(E\right)=\sqrt{\varepsilon_{\infty }}+\frac{(-B_{0}D-2C_{0})E+2B_{0}F+C_{0}D}{E^{2}-D\cdot E+F}$$

where the relations between the coefficients are:

(11) $$B_{0}=\frac{1}{Q}\cdot \left(B-2CE_{g}+CD\right)$$

(12) $$C_{0}=\frac{1}{Q}\cdot \left(A-BE_{g}+CE_{g}{}^{2}-CF\right)$$

(13) $$Q=\sqrt{4\cdot F-D^{2}}$$

Here, the condition $F-D^{2}>0$ must be met, and E_g_ and $\varepsilon_{\infty }$ have the same meaning as in the original Forouhi–Bloomer model.

Finally, in 2019, Forouhi and Bloomer [26] revised their original work and proposed a modification that is able to adequately fit experimental *n* and *k* data over wide ranges of the electromagnetic spectrum, either above or below E_g_. In addition, the new equations are consistent with the Principle of Causality. Considering that *k* shows only one peak, it can be expressed as:

(14) $$k\left(E\right)=\frac{A\cdot E}{E^{2}-B\cdot E+C}+\frac{A\cdot \left(E-E_{g}\right)}{E^{2}-B\cdot E+C}$$

where A is proportional to the probability for electron transitions, and in general to the probability of other types of transitions caused by photon interactions with phonons and other phenomena, such as quantized molecular rotational modes or mobile-proton transitions.

*n* can be described as:

(15) $$n\left(E\right)=\sqrt{\varepsilon_{\infty }}+\frac{D\prime \cdot E+F\prime }{E^{2}-B\cdot E+C}+\frac{D_{1}\cdot E+F_{1}}{E^{2}-B\cdot E+C}$$

where:

(16) $$D=\frac{A}{QQ_{1}}\cdot \left(E_{g}-\frac{B}{2}\right)$$

(17) $$F=\frac{A}{Q}\cdot \left(C-\frac{E_{g}B}{2}\right)$$

(18) $$D\prime =\frac{-\mathrm{AB}}{2Q}$$

(19) $$F\prime =\frac{AC}{Q}$$

A, B, C, D, F, D′ and F′ are constants related to the electronic structure of the material and E_g_ and $\varepsilon_{\infty }$ are the same as in the original FB model.

In order to correct inconsistencies of the original FB formulae, Jellison and Modine [35,36] proposed a model to describe the optical properties of amorphous materials. It is known as the Tauc–Lorentz model and combines the Tauc expression for the imaginary term of the dielectric constant near the band edge (ε_2_), with the imaginary part of the complex dielectric function for a single classical Lorenz oscillator. Despite this model being usually considered as mathematically correct, it is not completely analytical and still has several shortcomings that arise from not fully complying the conditions required for Kramers–Konig dispersion relations [33]. Furthermore, in the Tauc–Lorentz model, ε_2_ still becomes zero below the material bandgap energy. Consequently, it is not able to improve the modified FB model in this energy range.

Other models have been developed to incorporate the Urbach tail to the Tauc–Lorentz model. For instance, Likhachev et al. [37] proposed the Tauc–Lorentz–Lorentz–Gaussian model, in which additional unbounded Lorentz and Gaussian oscillators with transition energies located below the bandgap were included. This modified dispersion model can satisfactorily describe the absorption features below the bandgap and has enough flexibility to depict the dielectric functions for a wide variety of materials [37]. However, it retains the piecewise functions; hence, the absence of analyticity is not resolved. In this regard, Rodriguez-De Marcos et al. [33] reported a new method to transform the Tauc–Lorentz model into an analytic model and, subsequently, modified it to include the Urbach tail.

Pristine PEDOT:PSS thin films have an entirely amorphous structure [38] and are not only amorphous with direct bandgap as assumed in the original FB model but may exhibit indirect bandgap characteristic [39] such as phonon effects. Furthermore, the valence and conduction band edge of these thin films may consist of linear, parabolic and other type of band edges rather than the only parabolic part. Consequently, the optical properties of PEDOT:PSS need to be described by the modified FB models presented herein.

## 3. Results

In this section, the complex refractive indexes of seven PEDOT:PSS samples are described. Experimental results were taken from the articles reported by [12,18,19,20,21,22,23]. Both samples regarded as isotropic and others with optical anisotropy are included. For the anisotropic samples, the ordinary and the extraordinary components of the refractive index are shown. For all the samples, the real (*n*) and imaginary (*k*) parts of the refractive index were fitted to the original Forouhi–Bloomer equations [24], to the Forouhi–Bloomer 2019 revised formulae [26] and to the Lui equations [25]. What these three models have in common is that they are fully analytical and can accurately reproduce the experimental *n* and *k* values of the PEDOT:PSS samples described herein. Therefore, they fulfill a good trade-off between simplicity and precision. The values for the fitting parameters and the relative root mean square error (RMSE) for all the samples are reported below. In all the cases, the fit process was as follows:

- The fitting coefficients for *k* (A, B, C, D and E_g_, in the Liu model) were first calculated. They were obtained by minimizing the RMSE between the values calculated with the equations of the models and the experimental *k* data.
- The parameters were used to describe *n* (B_0_, C_0_ and Q) in the Liu model, and Equations (11)–(13), which depend on the coefficients for k, were then calculated.
- Finally, the fitting coefficient for *n* ($\varepsilon_{\infty }$ in the Liu model) was calculated, minimizing the RMSE between the values calculated with the theoretical equations and the experimental *n* data.

**Figure 1:**
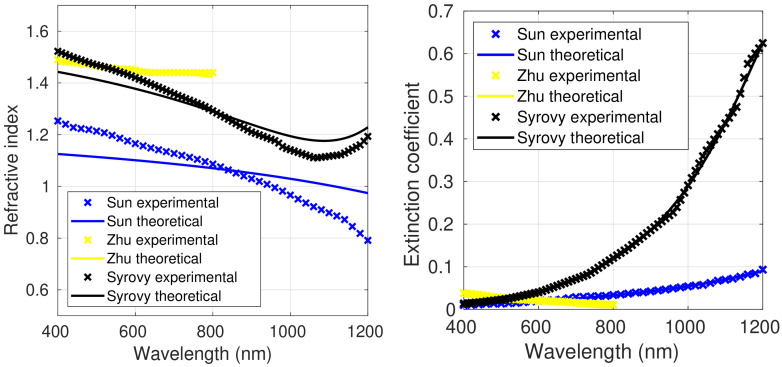
Refractive index and extinction coefficient of the three isotropic samples of PEDOT:PSS from [18,22,23]. The crosses correspond to the experimental values and the solid lines to the fit to the Liu model.

**Figure 2:**
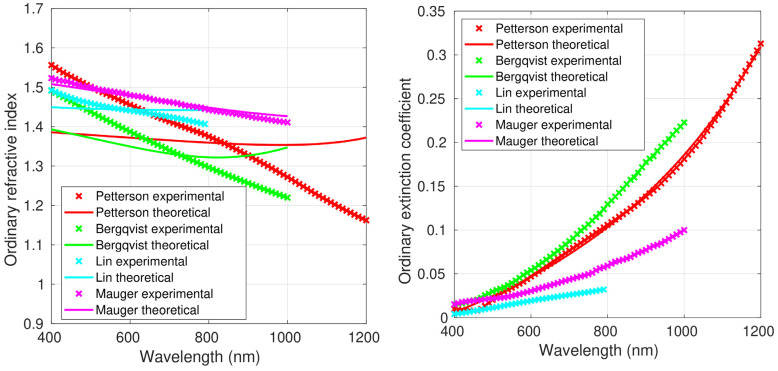
Ordinary component of the refractive index and extinction coefficient of the four anisotropic samples of PEDOT:PSS from [12,19,20,21]. The crosses correspond to the experimental values and the solid lines to the fit to the Liu model.

**Figure 3:**
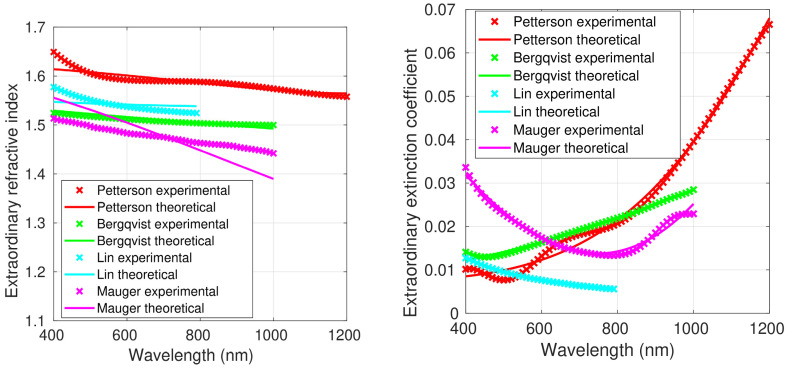
Extraordinary component of the refractive index and extinction coefficient of the four anisotropic samples of PEDOT:PSS from [12,19,20,21]. The crosses correspond to the experimental values and the solid lines to the fit to the Liu model.

Figure 1, Figure 2 and Figure 3 show both the experimental refractive index values and those obtained using the Liu model, which led to the smallest fitting errors, as shown below; thus, the figures with the fittings to the original FB model and the Forouhi–Bloomer 2019 model are not shown. Data for the optically isotropic samples are depicted in Figure 1 and for the optically anisotropic ones in Figure 2 (ordinary component of the complex refractive index) and Figure 3 (extraordinary component). Table 1 and Table 2 show the average values, standard deviations, minimum and maximum values of *n* and *k* in the spectral range between 400 and 800 nm for all the isotropic and anisotropic PEDOT:PSS films. The average *n* and *k* of all reported values are also included.

For all the samples, *n* laid within the range of 0.8–1.65. It decreased with increasing wavelength over the entire measured spectral range, showing normal dispersion behavior, except for the values reported by Syrovy et al. [22]. In this case, *n* increased for wavelengths above 1000 nm. The average *n* value (1.169) for the sample prepared by Sun and coworkers [23] was clearly lower than those of the other two isotropic samples (1.454 and 1.414 for those developed by Zhu et al. [18] and Syrovy et al. [22], respectively). This discrepancy cannot be attributed to the film thickness, since the former sample had approximately the same thickness (40 nm) as the one prepared by Zhu et al., while that developed by Syrovy and coworkers was considerably thicker (200 nm). A potential reason for the different behavior observed could be the different preparation method. Thus, Sun et al. prepared the films via spin coating while Zhu et al. and Syrovy et al. [22] via CFM and spiral bar coating, respectively. Another potential explanation for this discrepancy is the different morphology of the samples, since different deposition techniques result in diverse morphologies and, hence, different optical properties. Thus, for MAPbI3 perovskite films [40], it was reported that methods that generated a more agglomerated and compact structure led to smaller *n* values. In addition, the value obtained was conditioned by the measurement method; thus, SE with non-polarized light did provide information on the potential optical anisotropy of the material. These points will be discussed in a following section.

Except for the optically anisotropic film prepared by Mauger et al. [21], in which the ordinary and the extraordinary refractive indexes were very similar, the mean value of the extraordinary refractive index was between 7 and 10% higher than that of the ordinary component. The difference could be related to the film thickness, since the sample reported in [21] was considerably thinner than the others. On the other hand, the ordinary and extraordinary refractive indexes of the sample prepared by Lin et al. [20], with an average thickness of 100 nm, were very similar to the averages of the four anisotropic samples (see Table 1).

With regard to *k*, it was systematically below 0.35 except for the sample prepared by Syrovy et al. [22] that exceeded this value for wavelengths higher than 100 nm (Figure 1), reaching a value of about 0.6 at 1200 nm. Clearly, the average *k* value for the sample reported by Syrovy, 0.050, was higher than those of the other two isotropic samples (0.021 and 0.022 for those prepared by Sun et al. [23] and Zhu et al. [18], respectively). Probably, the higher *k* values found by Syrovy can be attributed to the significantly thicker films (200 nm vs. 40 nm). Thus, in the three isotropic films, the average *k* rose with increasing sample thickness. This trend was not observed in the anisotropic samples. In general, in the anisotropic films, the ordinary extinction coefficient was significantly higher than the extraordinary. The standard deviation was also significantly larger for the ordinary extinction coefficient, which accounts for its stronger change with the wavelength.

The influence of the thickness on the optical properties of thin films has been discussed previously in other works [41]. Some authors reported that the refractive index increased with increasing thickness (as found herein for the isotropic samples), due to an increase in the film density, as described in [42] for Al_2_O_3_. Similarly, Li et al. [15] found that *k* of Ta_2_O_5_ and *n* of ZnS, Ta_2_O_5_ and Al_2_O_3_ became higher with increasing thickness. In contrast, Han et al. [43] reported that *n* increased for thicknesses below 40–65 nm in polymers such as poly(2-vinyl pyridine) (P2VP), poly(methyl methacrylate) (PMMA), and polystyrene (PS) due to film inhomogeneities associated with non-uniform polarizability. Analogously, a drop in *n* of MgF [15] and in *k* of MnBi_0.95_Sb_0.05_ [41] was described. Other works showed the change in *n* with the film thickness, but no monotonic increasing or decreasing trend was found. For instance, Timoumi et al. [44] reported the change in E*g* and *n* of In_2_S_3_, but no clear trend was found. The same occurred for TiO_2_ in [15].

On the other hand, the average values of the ordinary and extraordinary extinction coefficients of the sample prepared by Pettersson et al. [19] were similar to the average of the four anisotropic samples investigated herein (0.014 and 0.040 for the ordinary and extraordinary component, respectively). However, the standard deviation of the values reported by Pettersson was significantly higher than that of the average of the four samples (Table 2).

In the following sections, the complex refractive index for each of the PEDOT:PSS samples is described in more detail. The influence of the preparation method, the model used to calculate the optical constants, the optical behavior and the film thickness is discussed. The fits of the complex refractive index to the original FB model, to the revised FB model (2019) and to the Liu model are also described, first for the isotropic films and then for the anisotropic ones.

### 3.1. Isotropic PEDOT:PSS Films

The PEDOT:PSS film studied by Sun et al. [23] was spin-coated on the front side of a silicon wafer. The film thickness was found to be about 40 nm. The optical properties were measured by spectral ellipsometry (SE) and non-polarized light (Table 3). The measurements did not reveal any optical anisotropy because non-polarized light is not able to detect it. However, it is expected that spectral ellipsometry measurements with polarized light would reveal optical uniaxial anisotropy in the sample, as a result of the axial symmetry of the film after being prepared by the spin coating method.

Figure 1 shows the experimental values for the complex refractive index reported by Sun et al. [23] and the theoretical values obtained by fitting to the Liu model. It can be observed that *n* decreased as the wavelength increased, from 1.25 at 400 nm to 0.8 at 1200 nm. These values were significantly lower than those obtained for the other two isotropic samples. A different behavior was found for *k*, which rose with increasing wavelength, from 0.01 at 400 nm to 0.09 at 1200 nm. The experimental values reported by Sun and coworkers [23] fit well to the three models. Table 4, Table 5 and Table 6 show the fitting parameters, and the RMSEs obtained are collected in Table 7. It can be observed that the best results were attained with the Liu model, with RMSEs as low as 2.97% for *k* and 7.81% for *n*. The values of the fitting coefficients D and F fulfilled the condition $4F-D^{2}>0$ (Table 6). The values obtained for E_g_ will be discussed below.

The PEDOT:PSS sample studied by Zhu et al. [18], with a thickness of around 40 nm, was prepared by capillary force molding (CFM). The optical properties were studied between 400 nm and 800 nm (Figure 1) using variable angle spectroscopic ellipsometry (VASE) and non-polarized light. No optical anisotropy was detected. In this sample, *n* remained approximately constant, at about 1.45, in the whole spectral range. On the other hand, *k* showed a decreasing trend with increasing wavelength, in contrast to the other isotropic samples. All the fitting parameters for the three models are shown in Table 4, Table 5 and Table 6. Very low RMSEs were obtained for all the fits (for instance, 0.44% for *n* and 3.18% for *k* with the Liu equations, Table 7).

The PEDOT:PSS film examined by Syrovy and coworkers [22] was prepared with the spiral bar coating method. The film thickness was about 200 nm. The optical properties were studied using SE and polarized light (Table 4). Although Syrovy et al. applied an anisotropic model to calculate the optical constants, the isotropic model worked better, since the film preparation method did not generate any symmetry that causes anisotropy. In this sample, the experimental *n* values decreased from 1.5 at 400 nm to about 1.15 at 1100 nm (Figure 1), while they increased slightly at higher wavelengths. In contrast, *k* showed an increasing behavior as the wavelength increased, with values between 0.01 and 0.6. This sample showed the highest *k* values among the three isotropic samples (Figure 1). It is important to note that this PEDOT:PSS film was significantly thicker than the others. The fitting coefficients obtained with the three models are compared in Table 4, Table 5 and Table 6. The best results for this sample were also obtained with the Liu model, with RMSEs as low as 3.54% and 3.81% for *n* and *k*, respectively (Table 7).

Table 7 summarizes the RMSEs of the fits to the three models for the three isotropic samples of PEDOT:PSS. As mentioned above, the best results were obtained with the Liu model, with RMSEs lower than 7.81% and 3.81% for *n* and *k*, respectively. In general, the FB model (2019) led to smaller errors than the original FB model.

### 3.2. Anisotropic PEDOT:PSS Films

The 110 nm thick PEDOT:PSS sample reported by Pettersson et al. [19] was prepared by means of the spin coating method. In this case, the material showed uniaxial anisotropic behavior, which was measured with VASE and polarized light. The complex indexes of refraction, parallel (ordinary) and perpendicular (extraordinary) to the surface plane of the films are displayed in Figure 2 and Figure 3. The ordinary refractive index decreased from 1.55 at 400 nm to 1.15 at 1200 nm. These values were very similar to those reported by Syrovy et al. [22]. The extraordinary refractive index remained almost constant with values ranging between 1.55 and 1.65. As shown above, the average values of the ordinary and extraordinary extinction coefficients of the sample prepared by Pettersson et al. were similar to the average of the four anisotropic samples. The ordinary extinction coefficient increased with increasing wavelength, reaching a value of around 0.30 at 1200 nm. The values of the extraordinary extinction coefficient were significantly lower than those of the ordinary component. They also rose with increasing wavelength, up to a value of about 0.07 at 1200 nm.

The experimental values reported by Pettersson et al. [19] fit well to the three models (Table 8, Table 9, Table 10 and Table 11). The best results were obtained with the Liu model, with RMSEs of 7.69% and 3.04% for the ordinary refractive index and extinction coefficient, respectively (Table 11). With regard to the extraordinary components, RMSEs of 0.51% and 4.68% were obtained for the two properties, respectively.

The PEDOT:PSS film prepared by Bergqvist et al. [12] was spin-coated on silicon substrates, and its thickness was 110 nm. SE measurements with polarized light were used for the optical characterization (Table 3). Figure 2 shows the ordinary refractive index and extinction coefficient in the spectral range of 400–1000 nm. The ordinary refractive index decreased from 1.5 at 400 nm to 1.20 at 1000 nm. These values were similar to those reported by Syrovy et al. [22] and by Pettersson et al. [19]. The ordinary extinction coefficient grew with increasing wavelength, reaching a value of around 0.23 at 1000 nm. RMSEs of 4.65% and 0.80% were obtained with the Liu model for the fits of the ordinary refractive index and extinction coefficient, respectively (Table 11). The extraordinary refractive index of the sample prepared by Bergqvist and coworkers remained almost constant at a value of 1.5 within the investigated wavelength range. The extraordinary extinction coefficient rose with increasing wavelength, up to a value of about 0.03 at 1000 nm. Similarly to the behavior found for Pettersson et al., the values of the extraordinary extinction coefficient were significantly lower than those of the ordinary component. RMSEs of 0.27% and 2.04% were obtained with the Liu model for the fit of the extraordinary refractive index and extinction coefficient, respectively. The fitting coefficients are shown in Table 10.

The PEDOT:PSS film reported by Lin et al. [20] was prepared using spin coating, with a thickness of 100 nm. The complex refractive index was determined using VASE with polarized light combined with the Drude–Lorentz model (Table 3). Similarly to the sample prepared by Zhu et al. [18], the ordinary refractive index remained approximately constant between 1.5 and 1.4 (Figure 2). The ordinary extinction coefficient grew as the wavelength increased, attaining a value of around 0.035 at 800 nm. As discussed above, the ordinary and extraordinary refractive indexes of the sample reported by Lin and coworkers were very similar to the averages of the four anisotropic samples. RMSEs as low as 1.44% for the ordinary refractive index and 0.95% for the ordinary extinction coefficient were obtained for the fit of this sample to the Liu model (Table 11). The extraordinary refractive index of this PEDOT:PSS film remained almost constant at a value of around 1.55, while the extraordinary extinction coefficient decreased with increasing wavelength, showing a maximum value of 0.012 at 400 nm. The fits to the Liu model gave RMSEs as low as 0.81% for the extraordinary refractive index and 0.27% for the extraordinary extinction coefficient (Table 10).

In the work by Mauger et al. [21], the PEDOT:PSS sample was spin-coated, and its thickness was measured to be about 40 nm. The complex refractive index was determined using VASE with polarized light combined with the Lorentz multiple oscillator model. The ordinary and extraordinary refractive indexes were quite similar, with values of around 1.5 (Figure 2 and Figure 3). The ordinary extinction coefficient rose as the wavelength increased, reaching a value of 0.1 at 1000 nm. Conversely, the extraordinary extinction coefficient decreased, from 0.035 at 400 nm to 0.013 at 800 nm. Very low RMSEs were obtained using the Liu model, 0.54% for the ordinary refractive index and 1.54% for the ordinary extinction coefficient (Table 11). The experimental values reported in [21] for the extraordinary refractive index and extinction coefficient fit well to the Liu model, with RMSEs as low as 2.03% and 3.74%, respectively (Table 11).

The energy bandgap calculated for each sample using the Liu model is shown in Table 6 and Table 10. E_g_ values between 0 and 2.1 eV were obtained. The average value for the isotropic samples was 2.13 eV, and the averages for the ordinary and the extraordinary components were 1.35 and 1.88 eV, respectively. These average values were within the range of those previously reported in the literature [11,12,20,45], which lay between 1.06 and 3.60 (Table 12).

It is important to note that the Liu model yielded a bandgap close to zero for some samples. However, it has to be taken into account that these were samples in which the material did not behave as a semiconductor, since the extinction coefficient was not zero in the whole spectral range. In this regard, no direct relationship between the bandgap and the high frequency refractive index, n_ꝏ_, was found, as predicted by the Moss rule (n_ꝏ_⁴ × Eg = 95 eV). Thus, the product n_ꝏ_⁴ × Eg was between 0.06 eV and 20 eV, far from the value postulated by the Moss rule of 95 eV. It should be noted that this rule is an approximation and it is only valid for semiconductors. Even in some semiconductors, this product has been found to be very far from the value predicted by the rule [46].

### 3.3. Application to the Simulation of Thin Film Multilayers

In this section, the influence of the differences in the measured values of the complex refractive index on the simulation of the optical properties of multilayers that contain PEDOT:PSS will be discussed. Specifically, it will be shown how these differences affect the ability of the material to absorb light. This issue is particularly important in the design of optoelectronic devices in which PEDOT:PSS acts as a transparent contact or as a hole transport layer like in OSCs. In such devices, the material must be highly transparent so that light is absorbed in the active layers. Therefore, the electrical parameters of the OSCs significantly depend on the refractive index of PEDOT:PSS and on its thickness.

The absorptivity of a multilayer composed of a PEDOT:PSS film on a glass substrate was calculated using the generalized matrix method proposed by Centurioni [16]. The refractive indexes of the three different isotropic samples and the average of these indexes were used for the calculations. A PEDOT:PSS thickness of 200 nm and a glass thickness of 1 mm were assumed.

**Figure 4:**
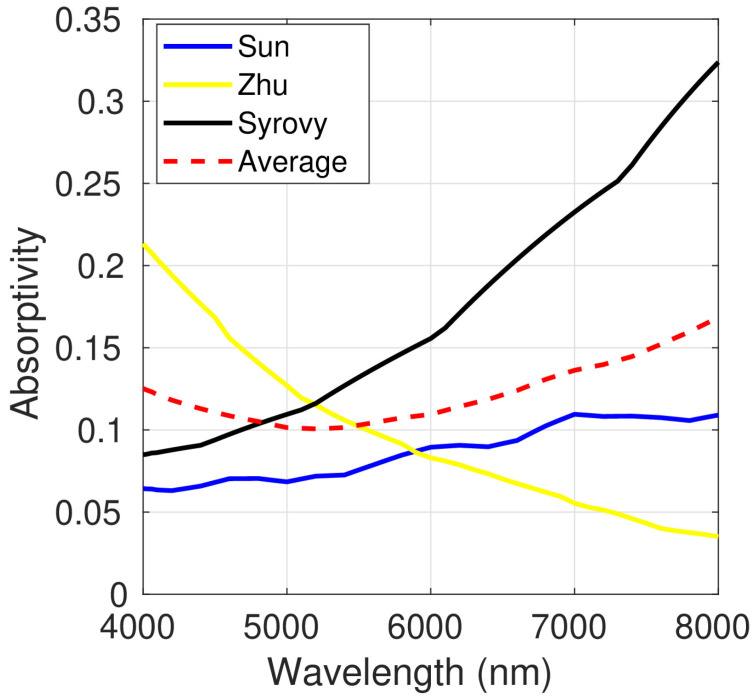
Absorptivity of a 200 nm thick PEDOT:PSS layer on a 1 mm glass substrate as a function of wavelength.

Figure 4 shows the calculated absorptivity in the wavelength range of 400–800 nm. The values obtained were significantly different for the three isotropic samples, regarding both the shape of the curve and the maximum and minimum values. As expected, higher values were obtained for the samples with higher extinction coefficient, as for the sample prepared by Syrovy et al. [22].

Since the absorptivity of PEDOT:PSS depends on the layer thickness, the average values in the range between 400 and 800 nm were calculated. The results are shown in Table 13, which includes the calculated values for PEDOT:PSS films with thicknesses of 20, 50, 100 and 200 nm. In all cases, the thickness of the glass was set at 1 mm. Noticeable differences were found depending on the refractive index used to make the calculations. Thus, the values obtained from the data reported by Sun et al. [23] (the most absorbent) were between 2 and 2.4-fold higher than those calculated using the refractive index described by Syrovy et al. [22] (the least absorbent). The smallest difference was found between the values calculated from the data reported by Sun et al. [23] and Zhu et al. [18]. However, this was only valid for the average values. The shape of the curve (Figure 4) revealed important changes in the absorption for almost all the spectral range investigated.

## 4. Conclusions

The complex refractive indexes of seven PEDOT:PSS samples, three with isotropic behavior and four with optical anisotropy have been described. Their differences in *n* and *k* with regard to the preparation method, the characterization method and the film thickness were discussed. For all the samples, *n* was within the range of 0.8–1.65, and it decreased with increasing wavelength over the whole spectral range below 1000 nm. Significant differences in the average *n* (up to 20%) were found. In most of the anisotropic films, the mean extraordinary refractive index was between 7 and 10% higher than the ordinary component. In the isotropic films, *k* rose with increasing film thickness, while this trend was not observed in the anisotropic, in which the ordinary extinction coefficient was significantly higher than the extraordinary. The standard deviation was also larger for the ordinary component, which accounts for its stronger change with the wavelength.

The real and imaginary parts of the refractive index were fitted to three models: the original FB model (1986), the Liu model (2007) and the revised version of the FB model (2019). RMSEs lower than 12% were obtained with the three models for all the films. Liu’s formulae give better results, with low RMSEs for both *n* and *k* (<7.81 and 4.68%, respectively, for all the samples). However, this model had two more fitting parameters, which led to significantly longer computation time than the other two models.

The influence of the differences in the complex refractive index on the simulation of the absorptivity of PEDOT:PSS multilayers was also discussed. The calculated average absorptivity strongly depended on the values of the refractive index used in the calculations. The absorptivities of the most absorbent sample were between 2 and 2.4-fold higher than those calculated using the refractive index of the least absorbent one. This clearly demonstrates the importance of measuring the refractive index of the material used in each specific research, or, as a lesser evil, to use values of a sample with similar thickness and manufactured with the same method. It is also essential to use characterization techniques that can detect optical anisotropy. Thus, the PEDOT:PSS films prepared by spin coating were found to be uniaxial anisotropic when a characterization method able to detect optical anisotropy was used. Finally, it is crucial to experimentally measure the change in the refractive index with the film thickness.

## Figures and Tables

**Table 1:** Sample thickness, average value, standard deviation (*σ*), minimum and maximum value of the refractive index in the spectral range between 400 and 800 nm for the isotropic and anisotropic PEDOT:PSS films. The averages of all reported values are also included.

Component	Thickness (nm)	Average *n*	*σ*_n_	*n* (600 nm)	Minimum *n*	Maximum *n*	Ref.
	40	1.169	0.051	1.166	1.086	1.253	[23]
	40	1.454	0.016	1.450	1.440	1.490	[18]
	200	1.414	0.068	1.421	1.292	1.522	[22]
Average		1.346	0.045		1.273	1.422	
Ordinary	110	1.459	0.053	1.455	1.375	1.556	[19]
Extraordinary	110	1.601	0.017	1.592	1.588	1.649	[19]
Ordinary	110	1.390	0.058	1.387	1.297	1.492	[12]
Extraordinary	110	1.513	0.006	1.512	1.504	1.524	[12]
Ordinary	100	1.444	0.023	1.441	1.407	1.493	[20]
Extraordinary	100	1.542	0.015	1.537	1.524	1.578	[20]
Ordinary	40	1.481	0.023	1.480	1.443	1.523	[21]
Extraordinary	40	1.486	0.015	1.483	1.463	1.514	[21]
Average Ordinary		1.443	0.040		1.380	1.516	
Average Extraordinary		1.535	0.013		1.520	1.566	

**Table 2:** Sample thickness, average value, standard deviation, minimum and maximum value of the extinction coefficient in the spectral range between 400 and 800 nm for the isotropic and anisotropic PEDOT:PSS films. The averages of all reported values are also included.

Component	Thickness (nm)	Average *k*	*σ_k_*	*k* (600 nm)	Minimum *k*	Maximum *k*	Ref.
	40	0.021	0.008	0.020	0.010	0.034	[23]
	40	0.022	0.008	0.021	0.011	0.038	[18]
	200	0.050	0.032	0.040	0.014	0.121	[22]
Average		0.031	0.011		0.021	0.055	
Ordinary	110	0.050	0.032	0.047	0.008	0.106	[19]
Extraordinary	110	0.014	0.005	0.013	0.008	0.021	[19]
Ordinary	110	0.060	0.035	0.054	0.015	0.130	[12]
Extraordinary	110	0.017	0.003	0.016	0.013	0.022	[12]
Ordinary	100	0.018	0.009	0.019	0.004	0.032	[20]
Extraordinary	100	0.008	0.002	0.008	0.006	0.013	[20]
Ordinary	40	0.033	0.013	0.030	0.015	0.059	[21]
Extraordinary	40	0.019	0.006	0.017	0.013	0.034	[21]
Average Ordinary		0.040	0.022		0.011	0.082	
Average Extraordinary		0.014	0.001		0.013	0.018	

**Table 3:** Preparation method, measurement method, fitting model, thickness and optical behavior of the seven PEDOT:PSS samples.

Preparation Method	Measurement Method	Polarized Light	Fitting Model	Thickness (nm)	Optical Behavior	Ref.
Spin coating	SE	No	-	40 nm	Isotropic	[23]
Capillary Force Molding	VASE	No	-	40 nm	Isotropic	[18]
Spiral bar coating	VASE	Linearly polarized	Lorentz multiple oscillator model	200 nm	Isotropic	[22]
Spin coating	VASE	Linearly polarized	-	110 nm	Uniaxial anisotropic	[19]
Spin coating	SE	Linearly polarized	Drude–Lorentz dispersion model	110 nm	Uniaxial anisotropic	[12]
Spin coating	VASE	Linearly polarized	Drude–Lorentz dispersion model	100 nm	Biaxial anisotropic	[20]
Spin coating	VASE	Linearly polarized	Lorentz multiple oscillator model	40 nm	Uniaxial anisotropic	[21]

**Table 4:** Coefficients of the fits to the original Forouhi–Bloomer model (1986) for the complex refractive index of the three isotropic PEDOT:PSS films. The coefficients for the average of the three samples are also shown.

A (eV)	B (eV)	C (eV^2^)	E_g_ (eV)	ε_ꝏ_	Ref.
0.0075	18	86	1.45	1.17	[23]
0.088	4.75	960	3.30	2.02	[18]
0.019	19.82	101	1.13	1.98	[22]
0.010	20.83	111	3.13	1.90	Average

**Table 5:** Coefficients of the fits to the modified Forouhi–Bloomer model (2019) for the complex refractive index of the three isotropic PEDOT:PSS films. The coefficients for the average of the three samples are also shown.

A (eV)	B (eV)	C (eV^2^)	E_g_ (eV)	ε_ꝏ_	Ref.
0.011	1.20	0.40	0.25	1.22	[23]
0.28	5.40	40.00	1.68	1.83	[18]
0.02	1.92	0.96	0.36	2.11	[22]
0.02	1.68	0.72	1.50	1.87	Average

**Table 6:** Coefficients of the fits to the Liu model for the complex refractive index of the three isotropic PEDOT:PSS films. The coefficients for the average of the three samples are also shown.

A (eV^2^)	B (eV)	C	D (eV)	F (eV^2^)	E_g_ (eV)	ε_ꝏ_	Ref.
0.0072	0.024	0.00	1.20	0.37	0.60	1.08	[23]
0.043	0.087	0.10	0.00	0.88	1.60	1.24	[18]
0.049	0.033	−0.02	1.73	0.80	1.07	1.24	[22]
0.11	0.017	0.07	0.27	0.20	2.13	1.32	Average

**Table 7:** RMSEs for the fits of *n* and *k* obtained with the three models for the three isotropic PEDOT:PSS films.

Forouhi–Bloomer		Forouhi–Bloomer (2019)		Liu 2007		
*n* RMSE	*k* RMSE	*n* RMSE	*k* RMSE	*n* RMSE	*k* RMSE	Ref.
11.96%	5.79%	10.29%	3.50%	7.81%	2.97%	[23]
0.83%	2.70%	1.18%	2.56%	0.44%	3.18%	[18]
8.22%	8.84%	6.66%	4.91%	3.54%	3.81%	[22]
3.41%	3.84%	2.93%	6.91%	3.00%	1.16%	Average

**Table 8:** Coefficients of the fits to the original Forouhi–Bloomer model for the complex refractive index of the four anisotropic PEDOT:PSS films. The coefficients for the average of the four samples are also shown.

Component	A (eV)	B (eV)	C (eV^2^)	E_g_ (eV)	ε_ꝏ_	Ref.
Ordinary	0.018	19.17	96	1.30	2.00	[19]
Extraordinary	0.0045	18.75	92	1.56	2.56	[19]
Ordinary	0.014	24	149	2.80	1.90	[12]
Extraordinary	0.0090	15	62	2.56	2.28	[12]
Ordinary	0.0027	29.50	231	2.50	2.09	[20]
Extraordinary	0.018	7.74	607	1.18	2.35	[20]
Ordinary	0.0075	23.13	143	1.45	2.18	[21]
Extraordinary	0.063	0.25	850	1.55	2.13	[21]
Ordinary	0.008	24	149	2.04	2.18	Average
Extraordinary	0.013	4	19	0.88	2.36	Average

**Table 9:** Coefficients of the fits to the modified Forouhi–Bloomer model (2019) for the complex refractive index of the four anisotropic PEDOT:PSS films. The coefficients for the average of the four samples are also shown.

Component	A (eV)	B (eV)	C (eV^2^)	E_g_ (eV)	ε_ꝏ_	Ref.
Ordinary	0.019	1.66	0.78	0.015	2.08	[19]
Extraordinary	0.007	1.67	0.73	1.34	2.55	[19]
Ordinary	0.012	2.31	1.46	0.036	1.92	[12]
Extraordinary	0.017	0.0063	0	0.006	2.29	[12]
Ordinary	0.0031	2.88	2.37	0.060	2.08	[20]
Extraordinary	0.46	0	182.00	0.97	2.18	[20]
Ordinary	0.013	1.68	0.85	0.21	2.24	[21]
Extraordinary	0.183	0	32.60	0	2.01	[21]
Ordinary	0.007	2.62	1.91	0.20	2.08	Average
Extraordinary	0.034	0	5.33	0	2.24	Average

**Table 10:** Coefficients of the fits to the Liu model (2007) for the complex refractive index of the four anisotropic PEDOT:PSS films. The coefficients for the average of the four samples are also shown.

Component	A (eV^2^)	B (eV)	C	D (eV)	F (eV^2^)	E_g_ (eV)	ε_ꝏ_	Ref.
Ordinary	0.091	0.016	−0.047	1.33	0.47	1.83	1.19	[19]
Extraordinary	0.024	0.017	0.008	1.43	0.64	2.08	1.28	[19]
Ordinary	0.073	0.021	−0.008	1.87	1.17	0.041	1.21	[12]
Extraordinary	0.028	0.004	0.008	0.056	0.11	0.021	1.25	[12]
Ordinary	0.15	0.017	−0.017	0.13	2.06	0.021	1.22	[20]
Extraordinary	0.13	0.051	0.064	1.11	64.04	0.011	1.23	[20]
Ordinary	0.067	0.011	0.008	1.21	0.60	1.62	1.25	[21]
Extraordinary	0.029	0.044	0.082	0.72	0.40	1.71	1.28	[21]
Ordinary	0.079	0	−0.010	1.70	1.20	1.35	1.23	Average
Extraordinary	0.13	0.0013	0.080	0.96	8.00	1.88	1.25	Average

**Table 11:** RMSEs for the fits of *n* and *k* obtained with the three models for the four anisotropic PEDOT:PSS samples.

	Forouhi–Bloomer		Forouhi–Bloomer (2019)		Liu 2007		
Component	*n* RMSE	*k* RMSE	*n* RMSE	*k* RMSE	*n* RMSE	*k* RMSE	Ref.
Ordinary	8.49%	11.08%	7.07%	6.85%	7.69%	3.04%	[19]
Extraordinary	1.22%	4.47%	1.21%	5.08%	0.51%	4.68%	[19]
Ordinary	6.98%	8.37%	6.52%	3.87%	4.65%	0.80%	[12]
Extraordinary	0.48%	2.76%	0.41%	3.30%	0.27%	2.04%	[12]
Ordinary	1.82%	7.78%	1.88%	4.83%	1.44%	0.95%	[20]
Extraordinary	1.00%	3.15%	1.02%	1.66%	0.81%	0.27%	[20]
Ordinary	2.75%	4.55%	1.81%	1.70%	0.54%	1.54%	[21]
Extraordinary	0.10%	5.41%	1.53%	22.58%	2.03%	3.74%	[21]
Ordinary	2.02%	11.62%	3.26%	3.47%	2.04%	1.74%	Average
Extraordinary	0.89%	7.35%	1.79%	8.73%	1.05%	2.42%	Average

**Table 12:** Energy band gap for different PEDOT:PSS samples obtained from the literature.

Eg (eV)	Ref.
1.06	[20]
1.55	[12]
3.60	[11]
3.38	[45] p type
3.10	[45] n type

**Table 13:** Average absorptivity in the spectral range 400–800 nm for the three isotropic films over a 1 mm thick glass substrate for the indicated film thicknesses. The absorptivity for the average of the three refractive indexes is also shown.

20 (nm)	50 (nm)	100 (nm)	200 (nm)	Ref.
0.008	0.018	0.041	0.087	[23]
0.011	0.025	0.051	0.096	[18]
0.018	0.044	0.089	0.174	[22]
0.012	0.03	0.062	0.122	Average

## Data Availability

The authors confirm that the data supporting the findings of this study are available within the article.
